# An Experimental Inquiry into the Nature of Gravelly and Calculous Concretions in the Human Subject; and the Effects of Alkaline and Acid Substances on Them, in and out of the Body

**Published:** 1806-10

**Authors:** Thomas Egan


### *An experimental Inquiry into the Nature of Gravelly and Calculous Concretions in the Human Subject; and the Effects of Alkaline and Acid Substances on them, in and out of the Body.* By THOMAS EGAN, *M.D. M.R.I.A.*

[ Continued from our laſt, pp. 221—229. ]

The whole of the 14th experiment strikes us strongly with a semblance of what probably passes, under similar circumstances, in nature; and reminds us of the danger attendant upon acid impregnations, more particularly at bed-time, when the urine, by many hours retention and quiet, has ample time to deposit its uric acid contents in the bladder. From it also we learn, that the temperature of the human body, in place of retarding or preventing (as might be expected *à priori)* these pernicious effects, rather promotes them, and that to a considerable degree.

But whilst we endeavour to establish this point, from practical observation as well as experiment, we seem to have entirely forgot that the urine itself is an acid liquor, and that therefore, if acids were so prejudicial, it is not probable that the provident wisdom of nature would commit the discharge of this necessary excretion to a fluid, which, by prematurely separating it within the body, would completely defeat the object of her humane attention. And would she not, in the infinity of her resources, dispose of it by some less objectionable emunctory?

I would, in the first place, observe, that though healthy urine manifests the properties of an acid liquor, it is in the very smallest possible degree; so much so, that though mentioned long since by Moraung, Coldevillars, and other surgeons, yet it was not, either chemically or medically, acknowledged to be so, until the time of Scheele, who finally established this point, as well as the nature of the prevailing acid. And, secondly, that nothing can be more erroneous than the opinion, which so long prevailed, that the phosphoric acid existed in it in a naked or uncombined state. It is now well established that it is only in that of a weak acidule, or acidulous phosphate of lime, very little short, indeed, of the point of saturation; and hence the weakness of its action as an acid liquor: for were it not for litmus, and some of the more delicate of the vegetable blues, we should have been, even to this day, ignorant of this property; so very feeble, indeed, that it will often not affect an infusion of red cabbage, whilst it turns with litmus, and sometimes, but feebly, with this most delicate of all acid tests. A single drop of phosphoric acid was added to one ounce of distilled water. Of this weak acid impregnation, one drop was sufficient to turn the infusion of litmus of as clear a red as the mineral acids do; whilst seven of urine manifested but very weak effects of acidity, and required some time to show any. If the urine, therefore, does not exceed its natural standard of acidity, we have nothing to apprehend. And here, indeed, we must again admire the wonderful wisdom of providence. The occasion (may I be allowed to say so?) required some chemical discrimination. It was necessary to carefully provide for the expulsion of the recrementitious part of the osseous fabric, which is very considerable, out of the system; but as this salt is insoluble in an aqueous vehicle, such as the urine, nothing more would be necessary to obviate this difficulty than a certain degree of super-saturation, or state of acidule, which would more effectually provide for its solubility and its elimination. But by going thus far, whilst it attended to one excretion only, it would have entirely forsaken its charge of another, committed also to this fluid; and by this degree of super-saturation, precipitate, retain in the system the uric acid, and occasion as frequent an occurrence of gravelly and calculous complaints, amongst mankind in general, as now occurs among the gouty. It therefore prudently formed that degree only of acidulous phosphate of lime, which, though insoluble out of the body, was sufficiently soluble when assisted by its temperature. Nay, even for wise purposes, it has given a degree of latitute to this temperature, which, though narrow and confined indeed, is sufficient for its purposes; but where it precisely terminates, I am not at present prepared to say, though so easily determined.

Let us now, for a moment, consider how far any morbid deviation from this healthy standard (which sometimes happens) may through light on this subject. The most considerable, that I am acquainted with, occurs in the instance of gouty urine rendered towards the decline of the paroxysms. A single drop of this, though in a turbid state, affects the vegetable blues with an energy equal, or perhaps superior, to that of the strongest acetous acid; and requires a very considerable increased proportion of lime water to decompose it, for obvious reasons. This we find always depositing, sometimes from the bladder itself, but generally before it has entirely parted with its natural temperature, a very large proportion of a reddish brick-dust-like sediment, (a welcome harbinger to gouty patients,)

ents,) gradually declining, and keeping pace with the alleviation of symptoms, and the progressive return of the urine to its natural degree of acidity. This sediment, Scheele, Bergman, and Fourcroy, consider of the uric acid kind: and so it (but in part only) undoubtedly is, being in a smaller proportion than they were aware of. For, considering that the enormous quantity, rendered in a few days, was incompatible with the known minute proportion of this acid matter in urine, I was determined to make the following experiment:—To a considerable quantity of it, desiccated and well edulcorated with distilled water, were added three ounces of a weak alkaline lixivium; which, after a few hours digestion, completely discoloured it, acquired a golden yellow colour, a sweetish taste, and, on the addition of a few drops of dilute marine acid, precipitated a copious sediment of whitish, minute, needle-shaped crystals, of a silky appearance.

To this precipitate, well edulcorated, was added, by degrees, about one ounce of weak nitrous acid, which acted on it with effervescence, and nearly took up the whole. This solution, being set to evaporate, began to redden the fingers, and other animal matters; no doubt, therefore, could subsist as to its nature. To the remainder, which seemed very little diminished, and only deprived of colour, were added two ounces of dilute marine acid, which, after some time in digestion, nearly dissolved the whole; and finding this acid solution precipitate with lime water, oxalate of ammonia, and fixed alkali, it must have been phosphate of lime. This forms, then, by far the largest proportion of the gouty sediment, which is coloured by the precipitated uric acid. Such also is the result of Crookshank's experiments; and so we should expect to find it, as I shall endeavour to point out, on a future occasion.

Let us now consider how far these analytical results may be confirmed in the synthetic way, having resolved that experiment, as far as applicable, should form the basis of any opinions offered in this essay. The phosphoric, being the native acid, prevalent in urine, it was interesting to determine, whether, by the artificial super-addition of it, so as to bring this fluid to the standard of the gouty, we might not produce effects somewhat analogous to what occur there.

Eighteen ounces of urine were divided into three equal parts. To the first were added five drops of sulphuric acid; to the second, ten; and to the third, fifteen. In the first, the magnifier very soon discovered minute floating moleculæ, gradually assuming the crystalline form, &c. as so often before described. In the second, the same appearances, but more immediately and copiously produced. But in the third, so considerable as to excite my astonishment. For here, besides the same extremely minute crystals which adhered to the entire sides of the phial, the bottom appeared covered with a mixture of crystalline and red pulverulent matter; the latter in great proportion, and probably prevented from crystallization by its hasty deposition. Here, then, that increased proportion of calcareous phosphate and animal gelatinous matters, (which always takes place in gout, and could not be expected here,) would seem only wanting to form a sort of synthetic approximation to the gouty sediment.

The unusual proportion of deposited uric acid in this experiment, created some suspicion that the phosphoric acid might, by a combination with some of the principles of this very compound fluid, give rise to some artificial formation of it on this occasion.

To the filtered liquor, therefore, of No. 3, were again super-added five drops, which in twenty-four hours caused a further separation of a very few crystals only. It was filtered a third time, and eight drops more added; but without the smallest appearance of a single crystal after four days. The additional acid, then, only more effectually and speedily determined the separation of the quantity naturally contained in urine; its more divided pulverulent appearance adding considerably to its volume.

It now only remained to demonstrate the identity of these various precipitates with the naturally deposited matter of gravel. For, though it could not be well mistaken for any other saline composition in urine; yet, as external characters are, even in the hands of a Romé de Lisle, or an Abbé Haüy, fallacious, the following, and concluding one, on the subject of acids was instituted.

Exp. 15.—To two drachms of this artificial gravelly matter was gradually added one ounce of nitrous acid, which acted on it with effervescence, and dissolved the whole, with the exception of some small, floating, succulent, animal particles, so well described by Bergman.

The evaporated solution reddened the skin, and, after some time, deposited crystals of oxalic acid; as happens in all concentrated nitrous solutions of calculi of the uric acid kind. To another small quantity was added some pure alkaline lixivium, which very soon took it up, became coloured, sweetish, and deposited the usual silky crystalline, line sediment upon the addition of acetous acid. No doubt, therefore, could remain, as to its identity with that naturally deposited.

And here, though irrelevant to my present object, and merely with a view to excite the attention of the faculty, may I be permitted to ask, how it happens, that in the very worst kinds of typhus fever there is very little diminution of the secretion or excretion of the acidulous phosphate of lime? as appears by the acidity of the urine, lime water, and the quantum of precipitate afforded by the oxalic acid; whilst a very considerable one of the uric acid takes place, and continues so until nearly the termination of the disease, when it begins gradually again to manifest itself; first, by the usual tests only, but presently, upon the crisis taking place, in such quantity as to become insoluble; and therefore quickly precipitates (with some additional mixture of calcareous phosphate and animal mucilaginous matter) under the form of our critical sediment or deposit? or, are we not here again to admire the wise œconomy of the Author of nature, which, by keeping up the considerable and necessary bony excretion of the system, prevents the dangerous accumulation of it which must ensue from its retention, during the long protracted period of many fevers? I might here offer some conjectures in explanation, but will reserve them for another place.

Having already trespassed so much upon the indulgence of the Reader, I shall here content myself with briefly stating, that, from the above experiments and observations, we may presume to say acids of every kind are prejudicial, and give rise to the formation of gravelly and calculous affections, by causing a separation and crystallization of the lithic acid contents of the urine within the body; not pretending, however, to deny the existence of other causes inherent in the system itself, occasionally productive of similar effects, as has been already observed.

I shall now proceed to the second part of this inquiry; namely, how far, or in what manner, alkaline matters are conducive to the alleviation of these complaints.

The bad effects of all acid and ascescent substances being generally felt and acknowledged, we cannot be surprised that sufferers from these maladies should naturally expect an alleviation of their complaints from substances of a very opposite nature; or that, perhaps, in the general anxiety of mankind to discover a solvent of these concretions, the active agency of alkaline matters could not be overlooked. We accordingly find, that from the remotest antiquity up to this day, they were, and still are, though under various modifications, chiefly resorted to.—Our ancient physicians prescribed waters with mineral alkaline impregnation, such as Seltzer, Carlsbad, and others; and, in latter times, we find our own countrymen more particularly engaged in these pursuits. Lime water, recommended by White, (to whose numerous and interesting experiments I must beg leave to refer); lime, and pure alkaline matter, forming the bases of the celebrated remedies of madam Stephens, Hartley, and others. And, in our own days, the caustic lixivium, again forgot, to make room for the more modern and fashionable introduction of both our alkaline sub and super carbonates; the vegetable, as in Faulkner's mephitic alkaline water, or in the crystallized carbonate of potash; the mineral, in a desiccated state, as recommended by my learned and indefatigable friend, Dr. Beddoes; or in that of the well known soda waters, first introduced in Geneva and Paris.

Now, in whatever of the above forms these saline matters are employed, their decided good effects are universally experienced and acknowledged. The aqua mephitica alkalina I consider the most valuable gift bestowed upon mankind by our modern chemistry; and to Beddoes's desiccated soda pills, my colleagues must join with me in acknowledging our greatest obligations. But how account for these good effects? or what can their modus operandi be? *Hic labor, hoc opus.* Carbonates, we have always been given to understand, exerted no solvent power on gravelly or calculous matter; and this continues to be the opinion of philosophers, as well as medical chemists, to this day. We find Fourcroy, in his late elaborate work on this subject, still continuing to assert, (in mentioning the action of various matters upon uric acid,) "les carbonates alkalines n'ont aucune action sur lui." Nor does the difficulty diminish with respect to the pure alkalies; for, in the stomach and primæ viæ, they must return again to either a carbonated or saponaceous state. My ingenious friend, and master in chemistry, Mr. William Higgins, (in the work already quoted) emphatically exclaims, "Why not at once give soap? why not turn our attention to the mild mineral alkali?" With regard to the common alkaline carbonates in use, it may be observed that the saturation is not complete; and that the uncombined portion of alkaline matter may still exert its specific powers observable by its detergent quality, as has been so long since well explained by Mr. Kirwan. This explanation, however, however, could not extend to the potassa carbonata, or crystallized vegetable alkali, lately introduced, and with equal success. May I be permitted (notwithstanding its use as a test, and non-deliquescence) to entertain some doubt of its complete saturation; for that, prepared with the most scrupulous care, still retains its alkaline taste, and acts with energy on the vegetable blues. The carbonic may probably be too weak an acid to entirely annul its alkaline property in any proportion that we can possibly unite them in the solid state. I am informed by Mr. Kirwan, however, that this can be effected; but the saturation is temporary, and continues only during its most recent state. This we now accomplish, by mechanical means, in the fluid one of our soda and other mineral waters; where, indeed, the alkali may be considered as in the super-carbonated state. Now, the success attendant also upon their exhibition, completely does away my former hypothesis; and we are left to conclude, that either the opinion of their want of action upon gravelly and calculous matter is unfounded, or that the animal œconomy may be possessed (among the multiplicity of its wonders) of some unknown chemical agency, whereby it may, in their course through the circulation, disengage their carbonic acid gas.

This would not appear more extraordinary than the formation of the different and most opposite secretions, such as bile and milk, from one and the same fluid; nor than what we every day observe to pass in the functions of the vegetable department of the organized kingdom. The salsola kali, salicornia, and other maritime plants, afforded to Chaptal's analysis, in their early state of growth, muriate of soda; when the plant was more advanced, this salt, with this excess of alkali; but in a full state of maturity, the same quite disengaged, and uncombined with muriatic acid. Here, then, we have one of our most refractory salts, and that resists the action of our greatest fires, completely decomposed by the vegetative powers of an humble plant. In this state of uncertainty I determined upon a course of some experiments which might throw some light on this subject; and go to explain how, or upon what principle, alkaline earths or carbonates become remedies in those complaints. And here, again, I must bring to our recollection, that whatever retains the uric acid in a state of solution whilst in the body, must prevent the formation of gravel or calculous matter of that kind.

And to begin with lime water, so generally prescribed

 since since the time of White. In this, the quantity of pure earth in solution (being only one grain in 700 of water) is so minute, and it is, withal, so readily decomposed, that we could not, à priori, expect much from its agency.

On conversing, however, with my friend Dr. Harvey on this subject, (of whose professional acumen it is unnecessary to make mention here,) he observed, with that strength of reasoning peculiarly his own: "Whatever may be the result of your future inquiries, can you, for a moment, imagine that physicians of the first eminence, and of all nations, would still consent to tread in the path of empiricism, by persevering in the use of this remedy, if they were not retained in it by the irresistible evidence of a successful practice and observation? or that the late Dr. Smyth, a gentleman of great discernment, and extensive knowledge, would, so generally and promiscuously, prescribe lime water in gout and gravel, if he were not satisfied of its efficacy, as well as of the great similarity of these complaints?

Exp. 1.—To four ounces of healthy urine was added one ounce of lime water. A similar quantity of urine was set aside as a standard, both in close vessels; temperature varying from 60 to 75 degrees, being in August, 1799. In the first, no sign of the slightest separation, or crystallization of uric acid, after three, five, or seven days. Some observable in the standard after the third day, which increased in quantity to the fifth.

Exp. 2.—To the same quantity of urine was added half an ounce only of lime water, with the same appearances as before. No sign of uric acid after several days. The standard deposited a few crystals on the third morning.

Exp. 3.—To the same quantity of urine were added two drachms only of lime water, which, though insufficient to neutralize the disengaged phosphoric acid, yet seemed as effectually to prevent the separation of uric acid as the greater quantities employed in the former experiments.

Exp. 4.—To three ounces of the urine of a child, six years old, still warm, and subject to deposit gravel upon cooling, were added two drachms of lime water, which effectually prevented all separation of this matter, whilst the standard precipitated copiously this saline substance after three hours.

( To be Continued. )

